# Nucleosome Free Regions in Yeast Promoters Result from Competitive Binding of Transcription Factors That Interact with Chromatin Modifiers

**DOI:** 10.1371/journal.pcbi.1003181

**Published:** 2013-08-22

**Authors:** Evgeniy A. Ozonov, Erik van Nimwegen

**Affiliations:** Biozentrum, University of Basel, and Swiss Institute of Bioinformatics, Basel, Switzerland; Ottawa University, Canada

## Abstract

Because DNA packaging in nucleosomes modulates its accessibility to transcription factors (TFs), unraveling the causal determinants of nucleosome positioning is of great importance to understanding gene regulation. Although there is evidence that intrinsic sequence specificity contributes to nucleosome positioning, the extent to which other factors contribute to nucleosome positioning is currently highly debated. Here we obtained both in vivo and in vitro reference maps of positions that are either consistently covered or free of nucleosomes across multiple experimental data-sets in Saccharomyces cerevisiae. We then systematically quantified the contribution of TF binding to nucleosome positiong using a rigorous statistical mechanics model in which TFs compete with nucleosomes for binding DNA. Our results reconcile previous seemingly conflicting results on the determinants of nucleosome positioning and provide a quantitative explanation for the difference between in vivo and in vitro positioning. On a genome-wide scale, nucleosome positioning is dominated by the phasing of nucleosome arrays over gene bodies, and their positioning is mainly determined by the intrinsic sequence preferences of nucleosomes. In contrast, larger nucleosome free regions in promoters, which likely have a much more significant impact on gene expression, are determined mainly by TF binding. Interestingly, of the 158 yeast TFs included in our modeling, we find that only 10–20 significantly contribute to inducing nucleosome-free regions, and these TFs are highly enriched for having direct interations with chromatin remodelers. Together our results imply that nucleosome free regions in yeast promoters results from the binding of a specific class of TFs that recruit chromatin remodelers.

## Author Summary

The DNA of all eukaryotic organisms is packaged into nucleosomes, which cover roughly  of the genome. As nucleosome positioning profoundly affects DNA accessibility to other DNA binding proteins such as transcription factors (TFs), it plays an important role in transcription regulation. However, to what extent nucleosome positioning is guided by intrinsic DNA sequence preferences of nucleosomes, and to what extent other DNA binding factors play a role, is currently highly debated. Here we use a rigorous biophysical model to systematically study the relative contributions of intrinsic sequence preferences and competitive binding of TFs to nucleosome positioning in yeast. We find that, on the one hand, the phasing of the many small spacers within dense nucleosome arrays that cover gene bodies are mainly determined by intrinsic sequence preferences. On the other hand, larger nucleosome free regions (NFRs) in promoters are explained predominantly by TF binding. Strikingly, we find that only 10–20 TFs make a significant contribution to explaining NFRs, and these TFs are highly enriched for directly interacting with chromatin modifiers. Thus, the picture that emerges is that binding by a specific class of TFs recruits chromatin modifiers which mediate local nucleosome expulsion.

## Introduction

The genomes of all eukaryotic organisms are packaged into nucleosomes, which are the fundamental units of chromatin, each composed of approximately 147 base pairs (bp) of DNA wrapped around a histone octamer. Recent developments in technologies for measuring chromatin marks by chromatin immunoprecipitation (ChIP) on microarrays (ChIP-Chip) or by sequencing (ChIP-seq) have enabled the construction of genome-wide maps of nucleosome positions and modifications at high resolution across various conditions. These experimental data have revealed that nucleosomes are not uniformly distributed across the genome but rather that transcription start and termination sites are relatively depleted of nucleosomes [1], [2]. Furthermore, nucleosome positioning has been shown to vary across physiological conditions [3].

It has long been accepted that nucleosomes have intrinsic sequence preferences which influence nucleosome positioning, e.g. [4]–[6]. At the same time, it has also long been known that barriers in the DNA can cause nucleosomes to be ‘statistically positioned’ relative to such barriers, introducing a periodic pattern of nucleosome occupancy on both sides of the barrier [7]. Given the fact that nucleosomes may cover more than 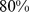 of the genome [1], it is therefore also conceivable that a relatively small number of barriers on the DNA, in combination with statistical positioning relative to these barriers, determines most of the observed nucleosome positioning. For example, recent work suggests that nucleosome occupancy patterns around TSSs could at least partly be explained by such statistical positioning [8].

Probably the most obvious class of candidate molecules that could introduce condition-specific barriers on the DNA are sequence-specific transcription factors (TFs). Indeed, for some specific promoters in *S. cerevisiae* it has been established that binding of TFs is a major determinant of nucleosome positioning in the promoter region, e.g. [9]–[11]. Moreover, the resulting nucleosome positioning has major effects on gene regulation from these promoters. In addition, for a few TFs it has been established that their binding induces local nucleosome exclusion genome-wide [1], [12]–[14].

Although it is thus clear that both intrinsic sequence preferences of nucleosomes and competitive binding of other DNA binding factors play a role in nucleosome positioning, the relative importance of these factors have come under intense debate in recent years. For example, it has been proposed that the positioning of nucleosomes, in particular in *S. cerevisiae*, is mainly determined by intrinsic sequence preference of the nucleosomes, i.e. [15]. In this view, nucleosomes are mainly positioned by a ‘code’ in the DNA sequence and the accessibility of the DNA to TFs is downstream of this sequence-guided nucleosome positioning. However, these conclusions were challenged by several studies which suggested nucleosome sequence specificity can only explain a modest fraction of nucleosome positioning, and that statistical positioning likely also plays an important role [1], [2], [16], [17]. More recently, several groups have undertaken further experimental investigations into this question, in particular by experimentally comparing nucleosome positioning *in vivo* and *in vitro* [18], [19]. Although there is general agreement that these experimental studies confirmed that both intrinsic sequence preferences and the competitive binding of TFs play a role in nucleosome positioning, different authors came to strikingly different, and often seemingly contradictory conclusions regarding which of these factors play a dominant role [20]–[24]. It is thus clear that, rather than lacking sufficient experimental data, the current challenge in furthering our understanding of the determinants of nucleosome positioning lies in the quantitative interpretation of this data.

Here we show that, by analyzing existing experimental data in combination with rigorous computational modeling, important novel insights can be gained that reconcile previous seemingly contradictory observations, and that suggest a new picture of the mechanisms regulating nucleosome positions. In particular, we use a biophysical model to quantitatively assess the role of TFs in determining nucleosome positioning in *S. cerevisiae*, to assess which aspects of nucleosome positioning TFs contribute to most, and to identify whether there are subsets of TFs that play a predominant roles in this process. *S. cerevisiae* is a particularly attractive system for such an analysis because extensive nucleosome positioning data are available, and because it is essentially the only organism in which sequence-specificities are available for the very large majority of TFs.

Rather than assuming that intrinsic sequence preferences determine nucleosome positioning and that TF binding occurs preferentially at those regions not covered by nucleosomes, or vice versa, assuming that TF binding sets boundaries in the DNA against which nucleosomes are statistically positioned, in our model the TF binding and nucleosome positioning patterns are determined by a dynamic competition of all TFs and nucleosomes for binding to the DNA. Our model incorporates both the sequence preferences of the nucleosomes and of all TFs in a thermodynamic setting, and rigorously calculates the resulting equilibrium occupancies genome-wide as a function of the concentrations of all TFs and the nucleosomes.

Using this model in combination with experimental data we find that TF binding makes a substantial contribution to nucleosome positioning but only at a specific subset of genomic positions. In particular, the linker regions between nucleosomes can be clearly divided into two classes based on their size: the large majority of linkers is small (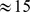 bp) and occurs within large nucleosome arrays in gene bodies, whereas a minority of linkers is large (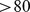 bp) and occurs predominantly in promoters. Our results show that the phasing of the small linkers within nucleosome arrays, and thereby the majority of nucleosome positioning genome-wide, is mainly determined by sequence preferences of nucleosomes. In contrast, the larger nucleosome free regions in promoters, which are likely most relevant for effects on gene expression, are mainly determined by competitive binding of TFs. By applying our model to data on nucleosome positioning *in vitro* we also confirm that the ability of TFs to explain nucleosome positioning in promoters is restricted to *in vivo* data. Thus, our model provides a quantitative and mechanistic explanation for the observed discrepancies between *in vivo* and *in vitro* nucleosome positioning. Most strikingly, our results also show that, rather than all TFs contributing roughly equally to the competition with nucleosomes, the effect of TFs on nucleosome positioning is restricted to a relatively small set of about 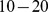 TFs. Although one might expect that these TFs are simply the highest expressed TFs with the largest number of TFBSs genome-wide in the conditions in which the experiments were performed, we find this not to be the case. Instead, we find that these TFs are highly enriched for having known protein-protein interactions with chromatin remodeling complexes, histones, and chromatin modification enzymes. Thus, the mechanistic picture suggested by our results is that there is a specific class of TFs who, upon binding to the DNA, recruit chromatin modifiers that then mediate local expulsion of nucleosomes.

## Results

### A biophysical model of TF and nucleosome binding to genomic DNA

To rigorously investigate the competition between TFs and nucleosomes for binding to DNA, and the role of TFs in nucleosome positioning, we take a statistical mechanics approach in which we explicitly consider all possible non-overlapping binding configurations to the genome for nucleosomes and a large set of TFs, assigning a probability to each configuration using standard Boltzmann-Gibbs statistics. The basic approach, which uses dynamic programming to efficiently sum over all possible binding configurations, has been used in computational methods for analysis of transcription regulation for over a decade, e.g. [17], [25]–[28], and has been used more recently to specifically investigate the effect of competitive binding of nucleosomes and TFs [29], [30]. Here we use this approach to comprehensively investigate the role of TFs in determining nucleosome positioning. We employ an unprecendented complete set of 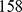 TF binding models, we investigate the dependence on the concentrations of these TFs, and we also introduce tunable sequence-specificities for all TFs and nucleosomes.

The model is explained in detail in the Materials and Methods. Briefly, each TF 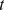 is assumed to bind DNA segments of a fixed length 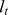 and, for any length-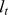 DNA segment 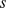, a binding energy 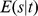 is determined. The energies 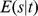 are calculated from a weight matrix representation of the TF's binding sites [31] and involve a tunable scale parameter 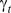 which controls the sequence-specificity of the TF. To obtain energy matrices for the large majority of sequence-specific TFs in *S. cerevisiae* we used a collection of 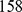 WMs that we curated previously [32] and that are based on a combination of ChIP-chip and *in vitro* binding data. Notably, while the WMs allow us to determine how the binding energy (measured in units 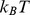) varies across positions in the genome for each TF, the WMs do not allow us to determine the sequence-independent contribution to binding energy, i.e. the overall ‘stickines’ of each TF for DNA. To compare binding energies across TFs we set the sequence-independent contribution to the binding energy such that all TFs have equal overall affinity for the DNA (see Materials and Methods).

**Figure 1:**
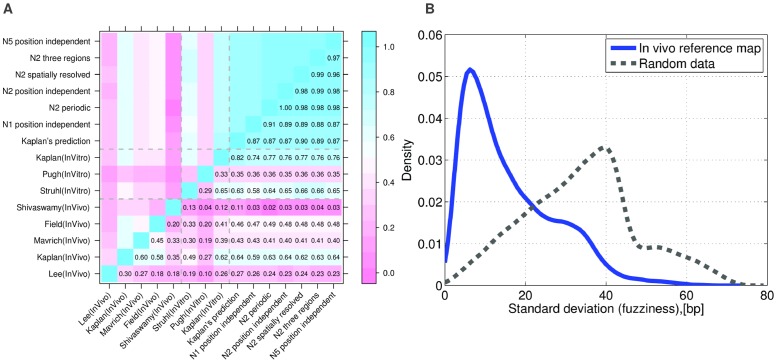
Reproducibility of *in vitro* and *in vivo* nucleosome data across different experiments and performance of nucleosome sequence-specificity models. **A:** Pearson correlation coefficients of the per-base nucleosome coverage between various experimental data-sets measuring nucleosome occupancy either *in vivo* [1], [3], [18], [38], [56] or *in vitro* [18], [19], [58], and predictions from a number of models of nucleosome sequence-specificity [18], [24]. **B:** Reproducibility of annotated nucleosome positions across the *in vivo* data-sets. For each annotated nucleosome in the reference map of [41], we calculated the standard deviation in the annotated positions of the corresponding nucleosomes across the 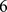 data-sets used to construct the map. The blue curve shows the distribution of standard deviations across nucleosomes. The grey dotted curve shows the analogous distribution that is obtained using randomized data (see Materials and Methods). The high reproducibility of nucleosome positions across different data-sets justifies the use of binary data, i.e. positions of “linkers” and “nucleosomes”, instead of Pearson correlation for evaluation of the performance of computational models for predicting nucleosome positions.

Of the computational work done on nucleosome positioning, probably most effort has been invested in developing models for nucleosome sequence-specificity based on data from both *in vivo* and *in vitro* nucleosome binding, e.g. [15], [18]. Exploiting analytical results from statistical mechanics, Locke et al. [24] rigorously inferred the energies of nucleosome binding from high-throughput data and used these to evaluate several models of different complexity for the sequence specificities of nucleosomes. The results from this study suggested that the sequence specificity of nucleosomes can be captured by fairly simple models. As we discuss below, our own analysis suggests that the performance of different models of nucleosome sequence specificity depends on the precise data-set and performance evaluation method used, but that all models make highly correlated predictions (Figure 1A). Of the models analyzed, the model of [18] gave robustly high performance across data-sets and we use this model in our study. In particular, we assume that nucleosomes bind to DNA segments of 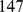 nucleotides and determine an energy of binding 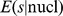 for any length 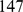 segment 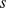 using a generalization of the model of [18], involving a scale parameter 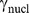 that controls the sequence specificity of the nucleosomes, analogous to the scale parameters 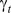 for the TFs (see Materials and Methods). The parameter 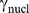 allows us to investigate the effect of enhancing or decreasing the nucleosome sequence specificity. For example, when setting 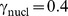, the variation in nucleosome binding energies across different sequences is reduced to 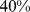 of the energy variations predicted by the model of [18].

As mentioned above, the model assumes that any DNA segment can only be bound by a single TF or a nucleosome at a time. Although it is likely that there are exceptions to this simplification, it is generally accepted that TFs and nucleosomes compete for binding to DNA. In absence of specific information as to which TFs compete with nucleosomes and which can co-bind with nucleosomes, we make the simplifying assumption that all TFs compete with nucleosomes, as has been done previously by others [29], [30]. Like previous approaches, e.g. [8], [15], [22], [29], our model also assumes that the average occupancy profiles across a population of cells are well approximated by their thermodynamic equilibrium averages. Notably, given that there are many ATP-driven processes that cause nucleosome turnover and displacement by chromatin remodelers, it is not a priori clear that this equilibrium assumption holds. Ours and previous computational approaches thus essentially assume that these ATP-driven processes act mainly to affect kinetics, i.e. to allow nucleosomes to resample their positions, without systematically biasing their positioning. Some recent evidence appears to support this assumption [33].

The model considers all possible non-overlapping configurations 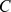 of TFs and nucleosomes bound along the genome. For each configuration 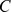, a total energy 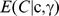 is calculated. This energy depends on the concentrations of nucleosomes 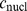 and all TFs 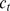, which we collectively denote as 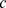, and also on all energy scale factors 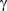 that determine sequence-specificity (Materials and Methods). The probability 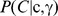 to find a cell in configuration 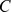 is then given by the standard Boltzmann-Gibbs formalism as
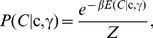
(1)where 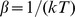 is the inverse temperature, 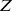 is the partition sum, and we have explicitly indicated that these probabilities depend on the concentrations 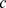 and scale factors 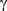. As explained in Materials and Methods, both the partition sum and the fractions of the time each TF 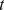 is bound at each genomic position can be calculated efficiently using standard dynamic programming techniques.

In summary, given a set of input concentrations 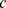 for all TFs and nucleosomes, the model efficiently calculates the equilibrium binding frequencies of all TFs and nucleosomes across the entire genome. Note that, because all TFs and nucleosomes are in competition for binding to the DNA, the occupancy of any factor to a sequence segment of the genome in principle depends, not only on the concentration of this factor and its affinity to the sequence segment, but on the concentrations of all other factors and their affinities to all other locations in the genome. Thus, the TF and nucleosome occupancy profiles across the genome can be changed by varying the concentrations 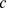 and scale factors 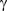. In particular, these parameters can be optimized to maximize the agreement with experimentally determined nucleosome occupancy profiles.

### Comparing model predictions with experimental nucleosome position profiles

Many experimental studies have been carried out to map nucleosome positions in eukaryotic species, e.g. [34]–[37], and in *Saccharomyces cerevisiae* in particular, e.g. [1]–[3], [18], [19], [38], [39], so that several data-sets of nucleosome positions in *S. cerevisiae* are available. In order to determine how to meaningfully compare computational predictions with these experimental data, we first performed a comparative analysis of several experimental data sets. Patterns of nucleosome positioning that are typically highlighted in publications, such as the nucleosome-depleted regions upstream of the transcription start sites (TSSs) and well-positioned nucleosomes immediately downstream of TSS, involve genome-wide averages of nucleosome occupancy across a class of positions. Such average patterns are robust to fluctuations and are shared by all data-sets.

Previous works have assessed the performance of models of nucleosome sequence specificity by determining both the predicted and experimentally observed nucleosome occupancies across individual regions of the genome, and by calculating the Pearson correlation of these nucleosome occupancy profiles. To assess the validity of such an approach, we calculated Pearson correlations between observed occupancy profiles of several experimental data-sets (both *in vivo* and *in vitro*) as well as several models of nucleosome sequence specificity (Figure 1A). This shows that, unfortunately, the occupancy profiles correlate only weakly across different experimental data-sets, with Pearson correlation coefficients typically ranging from 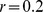 to 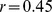 for *in vivo* data-sets, and only marginally higher for *in vitro* data-sets. This large variability across data-sets may to some extent be due to biases of the technological platforms. For example, it is well known that the nucleotide composition and propensity to form secondary structures of the reads can systematically bias the read counts in ChIP-seq by more than 10-fold [20], [40]. Variations in details of the ChIP protocol are likely also responsible for some of the variation across data-sets, and previous studies have indicated that MNase digestion bias may also systematically affect nucleosome positioning data [23], [24]. Since all experiments were performed in YPD, true biological variation is likely only a minor source of variation in these data.

In contrast to the experimental data, the occupancy profiles predicted by the different computational models are all highly correlated. Moreover, the correlations across models for a given data-set vary much less than the correlations for a given method vary across data-sets. For example, all models consistently perform better on *in vitro* than on *in vivo* data. Among the *in vivo* data-sets, all methods perform by far best on the *in vivo* data of Kaplan et al.[18] (which is also far more correlated with *in vitro* data than any other *in vivo* data-set) and far worst on the *in vivo* data of Shivaswamy et al. [3]. Thus, comparison of different models with existing data supports the conclusions of [24] that different models of nucleosome-specificity perform similarly in explaining nucleosome positioning. Since the model of Kaplan et al. [18] exhibits highest performance for the majority of *in vivo* and *in vitro* data-sets, we chose to use this model in our analysis. However, the weak correlation of nucleosome occupancy profiles across data-sets shows that assessing the performance of computational predictions by directly comparing predicted and observed nucleosome occupancies is highly problematic. A meaningful comparison of computational models requires that one first extracts those features of the nucleosome positioning that are reproducible across experimental data-sets.

In contrast to the absolute value of the ChIP signal, we observed that the positions of local maxima and minima in nucleosome occupancy are much better reproduced across data-sets. This reproducibility of the ‘peaks and troughs’ in the nucleosome occupancy profile has been observed previously [41], and has been used to create a reference set of ‘nucleosome’ and ‘linker’ segments. In this procedure, local maxima and minima are used to annotate nucleosomes and linkers in each data-set. These annotations are then intersected, with reference nucleosomes placed at the consensus positions of regions annotated as nucleosomes in all data-sets, and reference linkers the regions free of nucleosomes in all annotations. That the positions of annotated nucleosomes are highly reproducible across data-sets, especially compared to raw coverage and compared to nucleosome maps based on randomized data, is illustrated in Figure 1B. The annotated positions of individual nucleosomes across different data-sets typically vary by less than 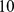 base pairs from the reference position (blue curve in Figure 1B) and the vast majority of annotated nucleosome positions vary by less than 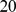 bp from the reference position. In contrast, on randomized data positions of annotated nucleosomes typically vary by roughly 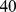 bp from the reference position (dotted curve in Figure 1B).

In summary, although ideally we would like to test whether computational models can predict relative nucleosome occupancies across the genome, it is not possible to meaningfully perform such an assessment given the variability observed in the experimental data. We thus evaluate the performance of different models by assessing their ability to predict nucleosome and linkers that occur consistently across different data-sets. We use the reference set annotated by [41] consisting of roughly 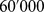 annotated linker regions and 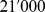 annotated nucleosomes, that together cover about 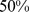 of the genome, to assess the performance of the model in predicting *in vivo* nucleosome positioning. In addition, we have applied a similar annotation procedure (Materials and Methods) to produce a reference set of nucleosomes and linkers from 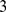 *in vitro* data-sets, which we use to assess the performance of the model in predicting nucleosome positioning *in vitro*.

To assess the model's performance we compare the predicted nucleosome coverage at annotated linker and nucleosome segments. That is, instead of comparing the predicted and observed absolute occupancies, we assess the model's ability to predict local maxima and minima in nucleosome occupancy, that occur consistently across data-sets. As described in Materials and Methods, based on the predicted nucleosome coverage, we classify each segment as either nucleosome or linker, and then calculate the *mutual information* 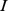 between the predicted and experimentally measured classification. Finally, we normalize this mutual information by the entropy 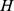 of the experimental classification to obtain the fraction 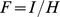 of information that is captured by the model's predictions, i.e. 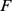 runs from 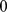 (random predictions) to 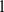 (perfect predictions). An 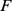 value of 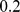 means that the model captures 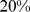 of all the information needed to specificy which of the genomic segments correspond to nucleosomes and which to linkers. We will refer 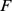 as the ‘quality score’. As mutual information is the fundamental measure of dependence between two distributions [42], [43], we consider the quality score 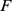 the most rigorous quantification of model performance. However, as we show below, highly similar results are obtained with other performance measures that are popular in machine learning, such as area under the ROC curve (AUC).

### Optimal fits to nucleosome positioning require weak nucleosome sequence specificity

**Figure 2:**
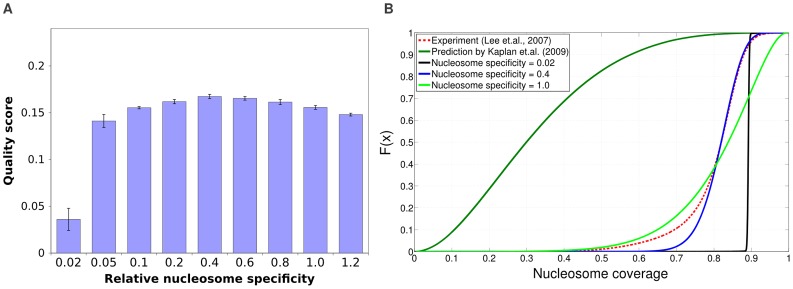
Performance of models that include only nucleosome sequence specificity. **A:** Fraction of information regarding experimentally annotated linker and nucleosome positions explained by the nucleosome-only model (quality score, vertical bars) as a function of relative nucleosome specificity. The relative nucleosome specificity is controlled by the scale factor 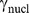, where 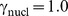 corresponds to the sequence specificity of the model of Kaplan et al. [18], for which the binding energy of the nucleosomes has a standard-deviation of 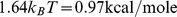 across the genome. The error-bars indicate standard-errors across 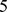 separate test sets. **B:** Experimentally observed cumulative distribution of nucleosome coverages (fraction of time a given genomic position is covered by a nucleosome) from [1] (red dotted line) and cumulative distributions of predicted nucleosome coverage of the models of [18] (dark green line) and our model using nucleosome specificity scale parameters of 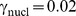 (black line), 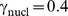 (blue line), and 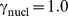 (light green line).

We first tested what quality score can be obtained by the intrinsic sequence specificity of the nucleosomes, i.e. leaving all TFs out of the model, and how the quality of the fit depends on the sequence specificity of the nucleosomes. Figure 2A shows the quality scores 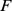 that are obtained for different scale factors 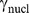 on nucleosome sequence specificity (with 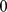 representing no sequence preference whatsoever and 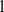 representing the specificity used in Kaplan et al. [18]). The optimal fit is obtained for 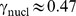, which corresponds to significantly lower nucleosome sequence specificity than those used in Kaplan et al. [18]. That is, for the model of [18], the standard deviation of nucleosome binding energies is approximately 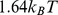 across the genome (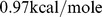), whereas we observe optimal fits for roughly 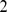-fold lower variations in binding energies (roughly 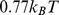). Moreover, the quality score depends weakly on 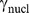 and becomes small only for extremely small sequence specificities.

These results may seem contradictory, given that the sequence-specificity model of Kaplan et al. was developed specifically with the aim of explaining nucleosome positioning. However, Kaplan et al. optimized the overall Pearson correlation between predicted and observed nucleosome coverage, which depends strongly on the variation in absolute nucleosome occupancies. In contrast, the quality score 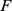 depends mainly on the locations of local maxima and minima in the occupancy, and much less on the absolute amount of variation in nucleosome occupancy. To investigate this further, we compared the distribution of nucleosome occupancies for the model with different values of 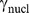 with the distribution of nucleosome occupancies for the model of Kaplan et al. and the experimentally observed distribution of nucleosome occupancies for the data of Lee et al. [1] (Materials and Methods, and note that very similar distributions are obtained from other experimental data-sets; Figure S1 in Text S1).

As shown in Figure 2B, the model of Kaplan et al. [18] predicts an overall nucleosome coverage that is dramatically lower than our fits, i.e. with a median nucleosome coverage of about 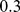. Such a coverage distribution is strongly at odds with the experimental data which shows that, rather than 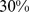, about 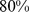 of the genome is covered by nucleosomes, e.g. [1], [3], [44], [45]. It is likely that the unrealistically low nucleosome occupancy of Kaplan et al. [18] is an artefact of optimizing the Pearson correlation in nucleosome coverage, since this objective function favors high variance in predicted nucleosome coverage, and does not penalize the mismatch in the average nucleosome coverage.

For our model, the coverage distribution indeed strongly depends on the nucleosome specificity. Strikingly, by far the best fit between the observed and predicted coverage distribution occurs precisely at the specificity that maximizes our quality score (i.e. at 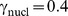). This demonstrates that, in contrast to the predictions of Kaplan et al. [18], our fits produce realistic nucleosome coverage profiles, in spite of not specifically optimizing these coverage profiles. In fact, at the optimal nucleosome specificity, the predicted and experimentally observed nucleosome coverage distribution is virtually identical for the 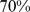 of base pairs in the genome with highest nucleosome coverage (blue and red curves in Figure 2B). The main deviation between model and experimental data is that the model fails to predict regions with low nucleosome coverage that are observed experimentally. Indeed, as we will see below, whereas the model correctly predicts almost all nucleosomes, the model fails to correctly predict a substantial fraction of linker regions as nucleosome free.

In summary, optimizing the quality score 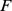 produces much more realistic fits to the nucleosome coverage distribution than previous models, and shows that the best fits are obtained with only weak nucleosome sequence-specificity.

### Transcription factor binding plays a major role in explaining nucleosome free regions at promoters

**Figure 3:**
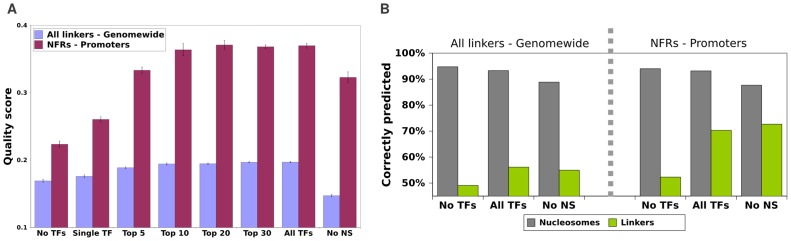
Incorporating competition with TFs improves predicted nucleosome positioning, particularly in promoter regions. **A**: Ability to predict nucleosome positioning as a function of the number of TFs used in the model. The bars show the fraction of all information regarding nucleosome positioning explained (quality score 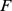) by each model. Results are shown for, from left to right, the model including only nucleosomes (no TFs), only the best TF, the top 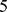 TFs, top 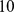 TFs, etcetera. The rightmost pair of bars correspond to a model including all TFs but without any sequence specificity for the nucleosomes 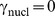. Blue bars correspond to quality scores for predicting all nucleosomes and linkers genome-wide and red bars correspond to quality scores for predicting nucleosomes and nucleosome free regions (long linkers) within promoters. The error bars show standard-error across 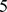 independent test-sets. **B**: Fractions of correctly predicted nucleosomes (grey bars) and linkers (green bars) for, from left to right, the model with nucleosome sequence specificity and no TFs, the model with all TFs, and the model with all TFs but no nucleosome sequence specificity. The left half of the figure shows results for predicting all linkers and nucleosome genome-wide, and the right half for predicting NFRs and nucleosomes in promoters.

We next investigated to what extent competition with TFs improves the predicted nucleosome positioning. We first considered models in which, besides the nucleosomes, there is only a single TF. For each of these models we fitted the 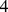 parameters (i.e. the concentrations and sequence specificity of both nucleosomes and the TF) using simulated annealing, and calculated the quality score 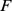 obtained with this model using 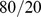 cross-validation (Materials and Methods). We ranked TFs by the 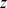-statistic they obtained in cross-validation (Materials and Methods), and then investigated what quality scores 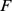 can be obtained using the top 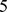, 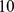, 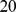 and top 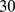 TFs, refitting all concentrations and sequence specificity parameters. We find that adding the TFs clearly increases the quality of the predictions on the test-sets, although the improvement is relatively small, i.e. from 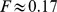 to 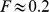, Figure 3A. Given this modest increase in 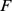 and the large number of parameters involved when including many TFs in parallel, one may wonder whether these results are affected by overfitting. However, as shown in Figure S2 in Text S1, the observed 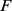 scores on train and test sets are essentially identical. In addition, adding the TFs to the model further improves the match between the observed and predicted nucleosome occupancy distribution (Figure S1 in Text S1).

As already observed in [41], the length distribution of linkers is bimodal. The large majority of linkers is short, around on average 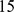 bps in length, corresponding to short linkers within arrays of nucleosomes. There is a second class, corresponding to roughly 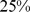 of all annotated linkers, that are much longer, i.e. each more than 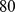 bps long. We will refer to these longer linkers as ‘nucleosome free regions’ (NFRs). We next asked whether TFs contribute more to explaining the positioning of the short linkers or the longer NFRs. Moreover, as TFs are expected to bind predominantly to promoter regions, we also investigated whether the contribution of the TFs to explaining nucleosome positioning is most significant in promoters (defined as running from 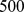 bp upstream to 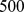 bp downstream of TSS). We find that, generally, inclusion of the TFs leads to a substantially larger increase in performance for promoter regions, and TFs contribute much more to explaining NFRs than explaining small linkers (Figure S3 in Text S1). In particular, considering NFRs and nucleosomes in promoter regions, inclusion of TFs almost doubles the quality score 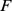, i.e. from 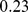 to 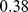, Figure 3A, red bars. As an aside, we note that these observations do not depend on assessing the model's performance by the quality score 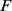. As shown in Figure S4 in Text S1, we find essentially the same results when assessing the model's performance using ROC curves, and the area under the curve (AUC) is almost perfectly correlated (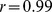) with the quality score 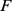. It is also noteworthy that, both when predicting all linkers genome-wide or NFRs in promoters, even though up to 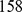 TFs can be incorporated, the model essentially reaches its optimal performance after adding the first 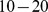 TFs. We investigate this in more detail below.

It thus appears that TFs contribute not so much to explaining positioned nucleosomes, but rather explain the location of longer NFRs, especially in promoters. Further supporting this observation, the rightmost pair of bars in Figure 3A shows the performance of the model including all TFs but with nucleosome sequence specificity removed, i.e. 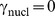. We see that removing nucleosome sequence specificity only modestly affects the ability of the model to predict NFRs in promoters. In contrast, the performance on predicting all linkers genome-wide drops significantly when nucleosome sequence specificity is removed, even falling clearly below the performance of the model without TFs. This is further confirmed by closer examination of the errors that the fitted models make (Figure 3B).

For all models, the large majority of nucleosomes is correctly predicted and the fraction of correctly predicted nucleosomes is most strongly affected by removing the sequence specificity of the nucleosomes, i.e. from 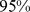 correct for the model with only nucleosome sequence specificity to 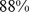 for the model with all TFs and no nucleosome specificity. The fraction of correctly predicted linkers is much smaller, e.g slightly below 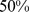 for the model without TFs. Adding the TFs to the model consistently increases the fraction of correctly predicted linkers, and this increase does not require nucleosome sequence specificity. When considering all linkers genome-wide, the increase in correctly predicted linkers is relatively modest, i.e. from 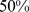 to 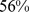. However, for NFRs in promoters the fraction of correctly predicted NFRs increases from 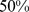 to around 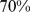. In summary, correctly predicting the phasing of nucleosome arrays over gene bodies crucially depends on nucleosome sequence specificity and is only weakly affected by including TFs, whereas correctly predicting NFRs is strongly dependent on inclusion of the TFs and is almost independent of nucleosome sequence specificity.

### Characterization and additional validation of the fitted model

**Figure 4:**
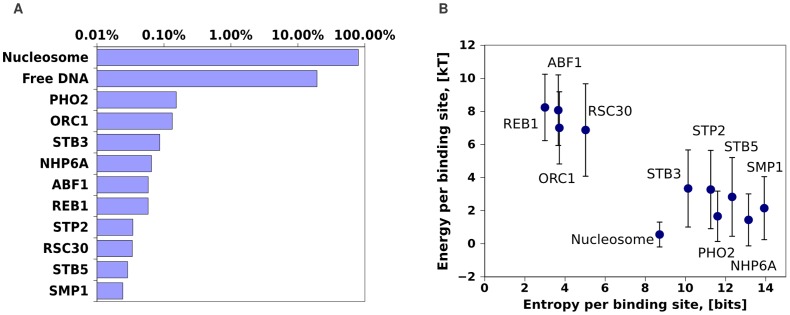
Biophysical properties of the fitted model. **A:** Average fraction of the genome covered by nucleosomes, free DNA, and the top 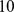 TFs with highest coverage. **B:** Average and standard-deviation of the binding energies (in units 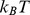) at binding sites for nucleosomes and the top 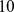 TFs with highest coverage (vertical axis), against the average entropy per binding site of the distribution of binding probabilities for the corresponding TFs (horizontal axis).

To characterize the biophysical properties of the fitted model we first determined the overall statistics of nucleosome and TF occupancies (Figure 4A). Nucleosomes cover more than 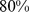 of the genome, and most of the remaining regions of the genome are uncovered, with all TFs combined covering less than 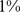 of the genome. The top 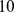 TFs with the highest genomic coverage occupy between 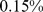 and 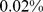 of the genome, corresponding to roughly 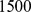 and 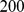 binding sites genome-wide.

For the nucleosomes and the top 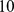 TFs with highest genomic coverage in the fitted model we also determined the mean and standard-deviation of the binding energies at their binding sites, and the entropy of the distribution of binding probabilities per site (Materials and Methods). The latter quantity is low whenever the TF's coverage results from strong sites with high frequencies of binding, and is high when the TF's coverage comes from a large set of weak sites with lower binding frequencies. The results (Figure 4) show, first of all, that the binding sites of nucleosomes have both the lowest binding energy and the lowest variation in binding energies, i.e. they are the least sequence specific. Interestingly, the top 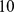 TFs clearly fall into 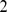 classes: a set of TFs (ABF1, REB1, ORC1, and RSC30) that are highly sequence specific and have strong binding sites, and a class of much less sequence specific TFs (PHO2, NHP6A, etcetera) that bind at a much larger number of weaker sites.

**Figure 5:**
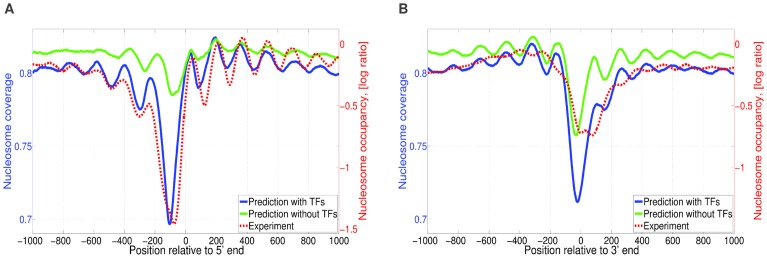
Predicted and observed nucleosome profiles around 5′ and 3′ ends of genes. **A**: Averaged nucleosome coverage near transcription starts. Each curve shows the average nucleosome coverage at different positions relative to transcription start averaged over all genes. Red dashed lines correspond to experimentally measured nucleosome coverage (data from [1], right vertical axis). The solid lines correspond to the predicted nucleosome coverage by the model including only nucleosomes (light green) and the model including all TFs (blue), left vertical axis. **B**: Averaged nucleosome coverage near transcription ends. Curves are as described for panel A.

As has been observed previously, e.g. [1], [2], averaged nucleosome coverage profiles show a characteristic pattern relative to the starts of genes with a nucleosome depleted region immediately upstream of TSS, followed by a well-positioned nucleosome immediately downstream of TSS and a periodic pattern of nucleosome coverage downstream into the gene body. Although the nucleosome sequence specificity by itself, i.e. without including TFs, reproduces some of this pattern at the 5′ end of genes (Figure 5A), the observed nucleosome depleted region and the oscillatory pattern into the gene body is much weaker than observed experimentally. As an additional test of the validity of our model, we checked whether inclusion of the TFs improves this average coverage profile relative to gene starts and ends.

We find that adding TFs to the model significantly improves the match between the theoretically predicted and experimentally observed nucleosome coverage pattern at the 5′ ends of genes (Figure 5A). It is noteworthy that the nucleosome-depleted region immediately upstream of TSS coincides with a peak in the overall predicted binding of TFs (Figure S5C in Text S1), further illustrating the role of TFs in establishing nucleosome depletion in these regions. A local peak in TF binding is also predicted immediately downstream of the 3′ ends of genes (Figure S5D in Text S1). Although at the 3′ ends of genes, the inclusion of the TFs also improves the match between the theoretical predictions and the experimentally observed nucleosome coverage, the experimental data and predictions clearly disagree (Figure 5B). First, the width of the experimentally observed NFR is twice as big as the width of the predicted NFR. Second, the oscillations exhibited by the experimentally-determined distribution are not as pronounced as predicted by the model. This lack of a match can likely be attributed to the role of RNA polymerase. Our model considers only 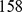 TFs and, in particular, does not consider the effects of binding of general transcription factors and RNA polymerase. Experimental data on the positioning of the largest subunit of Pol II - Rpo21, and the general transcription factor Sua7 shows that these factors localize at 3′ ends of genes [46], suggesting that they may contribute to the nucleosome free region observed at the 3′ ends of genes (Figure S6 in Text S1). This is further supported by the analysis in [47], which shows that rapid removal of Polymerase from 3′ end regions increases local nucleosome occupancy.

As another validation of the model, we investigated whether the predicted TF binding matches experimental observations. For example, we compared the intergenic regions predicted to be targeted by the TFs Abf1, Reb1, and Sum1, with the observed target intergenic regions according ot the ChIP-chip data of [48]. This shows that, in spite of the fact that the model was only optimized to fit nucleosome positioning, the fitted model also accurately predicts which regions are targeted by these TFs (Figure S7 in Text S1).

**Figure 6:**
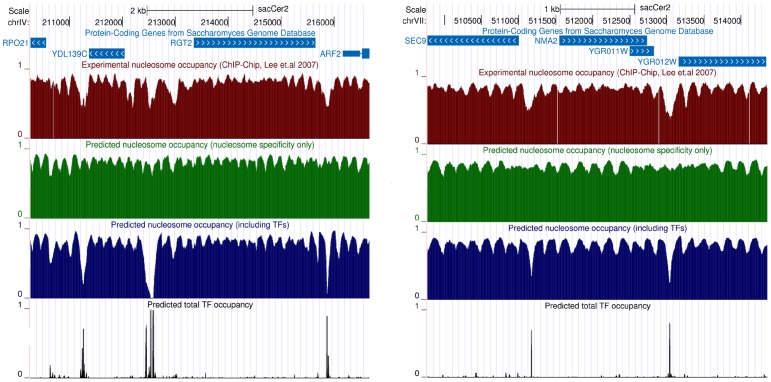
Illustration of the measured nucleosome occupancy and model predictions within individual genomic regions. Each panel shows a section of the yeast genome within our genome browser (swissregulon.unibas.ch/ozonov), with the tracks corresponding to, from top to bottom, chromosomal location, annotated genes, the measured nucleosome coverage based on the data from [1], the predicted nucleosome coverage using the model without TFs, the predicted nucleosome coverage using the model including TFs, and the total predicted TF coverage, i.e. summing over all TFs. Within the genome browser the coverage of individual TFs can also be displayed.

It is important to stress that, although we assess the model's performance by these global statistics, it predicts the precise locations of individual nucleosomes, NFRs, and TF binding sites. The full genome-wide nucleosome and TF coverage predictions obtained with the model including the TFs are made available through our SwissRegulon server www.swissregulon.unibas.ch/ozonov, allowing users to investigate in detail which NFRs at which promoters are explained by the binding of particular TFs. To illustrate the detailed comparison of the model's predictions and observed nucleosome occupancies Figure 6 shows the measured nucleosome coverage, the predictions of the model with and without TFs, and the predicted coverage of TFs, in two genomic regions. As the figure shows, whereas the locations of small peaks and troughs in occupancy across arrays of nucleosomes are reasonably well captured by nucleosome sequence specificity alone, competition with TF binding is needed to explain the occurrence of larger nucleosome free regions, which occur predominantly in promoters. Importantly, it is likely precisely this latter class of regions that are crucial for the effects of nucleosome positioning on gene expression.

However, this detailed comparison also reveals that, whereas the locations of TF binding typically matches the centers of observed NFRs, the predicted shape of these NFRs differs considerably between the model and the experimental observations. In particular, NFRs tend to be much narrower in the model's predictions than in the experimental data. This suggests that, although TF binding determines the genomic location where nucleosome depletion is observed, the observed nucleosome exclusion is more substantial than predicted from the steric hindrance between TFs and nucleosomes. This suggests that TF binding may recruit aditional factors involved in nucleosome exclusion. We return to this observation below.

### Only a small subset of TFs, enriched for interacting with chromatin modifiers, crucially affects nucleosome positioning

Our model incorporates the role of TFs through a simple competition for binding DNA and one might thus naively expect that all TFs that are expressed in YPD would contribute similarly to explaining nucleosome positioning, maybe in proportion to the number of their binding sites in the genome. However, we observed above (Figure 3A) that when consecutively adding more TFs to the model, the performance already assymptotes after 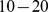 TFs. This could be due to redundancies in the contributions of the TFs, i.e. if sites for different TFs cluster in particular genomic regions, then binding by only a subset of the TFs will suffice to explain the occurrence of NFRs in these regions, and adding more TFs to the model would not further improve performance. Alternatively, it may be that there is a specific class of TFs that contribute much more to nucleosome positioning than other TFs.

**Figure 7:**
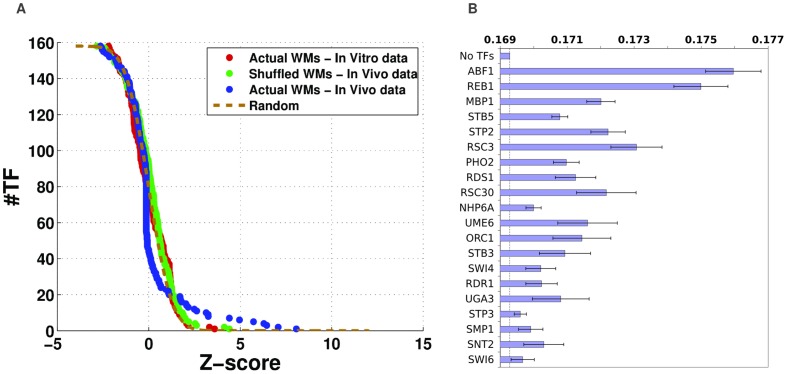
Only approximately 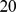 TFs contribute significantly to nucleosome positioning. **A**: For each TF an average quality score 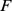 across 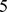 test-sets was determined using the model containing nucleosomes and the corresponding TF. TFs were then ordered by the 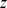-statistic 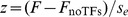, with 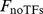 the quality score of the model without any TFs, and 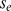 the standard-error across the 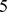 test-sets (see Materials and Methods). The panel shows the reverse cumulative distribution of 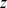-statistics observed across the 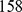 TFs (blue dots) together with the expected standard-normal distribution expected for random predictions (brown dotted curve). Note that about 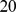 TFs have 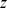-statistics larger than expected by chance. The green dots show the reverse-cumulatives of 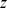-statistics for the fits obtained with WMs in which the columns of each WM have been randomly shuffled. The red dots show the reverse-cumulatives of 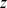-statistics obtained when fitting the original WMs to the *in vitro* map of nucleosome positions. Note that both the green and red dots closely follow the distribution expected by chance. **B**: The top 20 TFs that contribute most to *in vivo* nucleosome positioning sorted by their 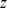-statistic. The bars show the average quality score 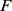 and standard-error 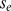 for each TF.

To investigate this, we used 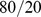 cross-validation on 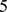 independent training and test sets to assess, for each of the 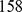 TFs, whether a model containing only nucleosomes and the single TF statistically significantly outperforms the model with only nucleosome specificity, quantifying the significance by a 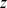-statistic (Materials and Methods). Figure 7A shows the distribution of 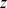-statistics obtained for the 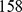 TFs (blue dots), together with the distribution of 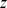-statistics expected by chance (brown dotted curve). As the figure shows, only 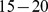 of the TFs significantly improve the predictions, indicating that there is indeed a specific class of TFs that dominate in explaining NFRs. Indeed, the large majority of all other TFs obtain quality scores on the test sets that are either the same or worse than the model without any TFs (Figure S8 in Text S1).

As another validation, we checked whether the ability of this subset of TFs to explain nucleosome positioning is a specific property of the sequence specificities of yeast's TFs. That is, it is in principle conceivable that among *any* set of WMs with similar information content and sequence composition, a few will be able to help explain nucleosome positioning. To test this we constructed a set of synthetic WMs by randomly shuffling the columns of the original WMs, and fitted models with these 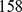 TFs in exact analogy to our fits with the original WMs. As shown in Figure 7A (green dots), none of the shuffled WMs perform better than expected by chance, confirming that the ability to explain nucleosome positioning is unique to the specific set of 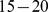 yeast WMs that we identified.

As a final test, we also evaluated whether the real WMs can explain the nucleosome positioning that is observed *in vitro* (Materials and Methods). On the one hand, since no TFs are present in the conditions at which the *in vitro* experiments are performed, the TFs should in principle not contribute to nucleosome positioning. On the other hand, as the raw *in vivo* and *in vitro* occupancies are significantly correlated (Figure 1A), one might expect that the TF WMs can still positively contribute to explaining *in vitro* nucleosome positioning. It is thus striking that none of the real yeast WMs performs better than expected by chance in explaining *in vitro* nucleosome positioning (Figure 7A, red dots), i.e. including TFs does not help explaining *in vitro* nucleosome positioning. This shows that the actions of a specific set of 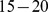 TFs are crucial for explaining the differences between *in vivo* and *in vitro* nucleosome occupancies.

Figure 7B lists the top 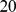 TFs and shows their quality scores on the test sets (results for all TFs are shown in Table S1). The fact that only around 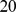 TFs contribute significantly to nucleosome positioning raises the question of what distinguishes these TFs from the others and we investigated a number of hypotheses. One might hypothesize that the top TFs are simply those that are highest expressed in YPD, or those which occupy most sites genome-wide. However, expression data indicates that these TFs are not particularly highly expressed in YPD compared to other TFs (Figure S9 in Text S1, data from [49]). Consistent with this, the genome-wide number of binding sites, as observed in genome-wide ChIP-chip experiments (Figure S10 in Text S1), is not generally higher for these TFs. Thus, the role of these TFs in nucleosome positioning is not simply the result of increased binding or expression in YPD. Notably, for a considerable number of TFs our model predicts essentially no binding sites, and not all of these TFs are low expressed in YPD. It is conceivable that the low number of predicted sites for these TFs indicates that these TFs do not compete with nucleosomes but can bind to DNA which is wrapped around a nucleosome. We also investigated whether the top 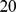 TFs have particularly high or low information content and found that this is not the case (Figure S11 in Text S1).

However, when we manually inspected the functional annotation of the top 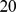 TFs, we noticed that roughly half of these TFs are known to be involved in chromatin remodeling (Table S1). Since, among our 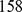 TFs only 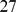 have been previously implicated in chromatin remodeling or nucleosome positioning, this amounts to a highly significant enrichment among our top 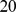 TFs (p-value 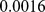, see Materials and Methods). This suggested that the top 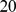 TFs may be characterized by interacting directly with chromatin modification machinery. To investigate this more systematically we investigated the occurrence of known direct protein-protein interactions between TFs and

1. Histones
2. Enzymes that modify histones
3. Proteins that are subunits of chromatin remodeling complexes

(see Materials and Methods). As detailed in Table 1, we find that our top 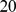 TFs are highly significantly enriched for direct protein-protein interactions with all 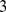 categories, showing the strongest enrichment for interacting directly with proteins in chromatin remodeling complexes. These results strongly suggest that our top 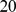 TFs are characterized by their ability to locally recruit chromatin modifiers.

**Table 1:** Statistical analysis of protein-protein interactions between TFs and chromatin remodeling complexes, histone modification enzymes, and histones.

Class	Total links	Links among top 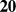 TFs	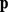 -value	Enrichment
Chromatin remodeler complexes	287	77	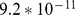	3.26
Histone modification enzymes	369	74	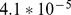	1.58
Histones	103	34	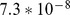	2.6
All three classes	718	176	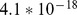	1.94

For all yeast TFs we counted the number of ‘links’, i.e. known direct protein-protein interactions, with proteins from the functional categories shown in the first column. The second column shows the total number of links with all TFs, and the third column the number of links with the top 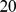 TFs that most significantly explain nucleosome positioning. The fourth column shows the 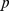-value for the enrichment of links among the top 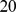 TFs using a hypergeometric test, and the 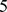 column shows the fold enrichment.

The fact that only those TFs that interact directly with chromatin modifiers contribute significantly to explaining NFRs has interesting implications for the mechanisms of nucleosome positioning. It suggests that the creation of NFRs depends on the actions of chromatin modifiers whose activities lead to local expulsion of nucleosomes from the DNA. That is, the mechanistic picture that emerges is that, initially, the competition between TFs and nucleosomes for binding DNA, as implemented in our model, determines where TFs will end up binding DNA. Subsequently, in those places where TFs from the specific class that can recruit chromatin modifiers are bound, the recruitment of these modifiers will lead to local expulsion of the nucleosomes, leaving a larger region depleted of nucleosomes. This mechanistic picture also explains our previous observation that the predicted NFRs tend to be much narrower than those observed in the data.

## Discussion

It is generally accepted that the packaging of DNA by nucleosomes in eukaryotes can modulate the accessibility of TFs to their cognate sites and thereby have major effects on gene regulation. In recent years there have been significant experimental efforts to determine nucleosome positioning patterns genome-wide, and to analyzing how these nucleosome-positioning patterns are established. As we discussed in the introduction, there has been a considerable debate as to whether nucleosome positioning in *Saccharomyces cerevisiae* is predominantly controlled by intrinsic sequence specificity of the nucleosomes, or that statistical positioning around barriers introduced by other DNA binding factors is more important for nucleosome positioning, and different researchers have presented seemingly contradictory results in this regard. We feel that these apparent contradictions may be reconciled by the results presented here.

The large majority of annotated nucleosomes and linkers genome-wide concern the phasing of short linkers within dense arrays of nucleosomes, mainly inside genes. We find that the positioning of these nucleosomes and short linkers crucially depends on the sequence specificity of the nucleosomes, and that TFs contribute relatively little to their positioning. Therefore, predicting all linkers and nucleosomes on a genome-wide scale, the sequence specificity of the nucleosomes provides the main contribution to explaining their positions. In contrast, we find that nucleosome specificity contributes little to explaining larger nucleosome free regions, especially those within promoter regions. As our modeling shows, NFRs in promoters are predominantly explained by the DNA binding of a specific class of 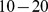 transcription factors. Thus, while genome-wide locations of nucleosomes and short linkers are predominantly determined by nucleosome sequence-specificity, the large nucleosome free regions in promoters that likely contribute much more significantly to gene regulation, are determined mainly through the competitive binding of TFs. Importantly, the fact that competition with TFs can not help explain the *in vitro* nucleosome positioning shows that the contributions of the TFs is restricted to *in vivo* positioning. Thus, the competitive binding of TFs provides a quantitative and mechanistic explanation for the differences between *in vivo* and *in vitro* nucleosome occupancies.

That nucleosome free regions in promoters result from a competition between TF and nucleosome binding is supported by a number of recent studies of individual promoters, e.g. [9]–[11], [50]. In these studies the interplay of TF and nucleosome binding determines positions of NFRs and the resulting accessibility pattern has major consequences for gene expression. Our results suggest that this mechanism is not restricted to a few promoters, but is the typical situation genome-wide. Thus, whereas nucleosome sequence specificity does have a major impact on genome-wide nucleosome positioning, precisely those aspects of nucleosome positioning that have most impact on gene regulation are rather determined by the competition between nucleosomes and TF binding.

Another major result from our study is that less than 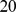 of the 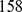 TFs that we analyzed appear to have a significant effect on nucleosome positioning. As we have shown, these TFs are not characterized by particularly high expression or large numbers of binding sites in YPD, nor do they possess particular sequence specificities or DNA binding domains. Instead, our analysis suggests that these TFs engage in specific protein-protein interactions with chromatin remodelers, thereby effecting nucleosome eviction much more dramatically than other TFs.

Although the final predictions of our statistical mechanical model are quite competent, i.e. in promoters 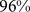 of all nucleosomes and 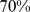 of all NFRs are correctly identified, they are still far from perfect. This raises the question as to what additional elements are missing from the model. The main error the model makes is failing to identify roughly one third of nucleosome free regions as nucleosome free. This suggests that the model misses additional factors that promote displacement of nucleosomes. As most sequence-specific TFs in yeast are already represented in the model, and our results suggest that only a small fraction of these TFs significantly affect nucleosome positioning, it seems unlikely that the missing sequence-specific TFs play a major role in the overall quality of the results. In contrast, as shown in Figure S6 in Text S1, general TFs including the RNA polymerase itself may play an important role in nucleosome positioning. In this context it has also been suggested [19] that the well-positioned nucleosome immediately downstream of TSS may result from a direct interaction between general transcription factors and the RNA polymerase with this nucleosome. Thus, including the recruitment and binding of general TFs and RNA polymerase will likely further improve the model.

In addition, TF binding can recruit chromatin modifying enzymes that displace nucleosomes and alter histone tails. The fact that experimentally observed NFRs are typically wider than the theoretically predicted ones suggest that the TF binding recruits chromatin modifiers which lead to a larger region of nucleosome exclusion than given by the TF binding itself. Thus, feed-back from TF binding to nucleosome modification and ejection as mediated by chromatin remodelers is a major feature that could improve the model's predictions. In summary, the picture that emerges from our study is that the binding of a specific class of 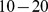 TFs determines local recruitment of chromatin remodelers, which then mediate local expulsion of nucleosomes. The latter may further positively feed-back on TF binding and thereby expand and stabilize the nucleosome-free regions.

Although this work has focused on yeast, the competition between nucleosomes and TFs for binding DNA may even be more crucial for transcription regulation in higher eukaryotes. For example, in multi-cellular eukaryotes many gene regulatory elements occur in distal enhancers, i.e. local clusters of TF binding sites a few hundred base pairs in length, to which a combination of TFs binds to effect transcription at a promoter that can be hundreds of kilobases away. Recent mapping of enhancers based on chromatin marks has suggested that these enhancers are bound and activated in a highly tissue- and condition-specific manner [51], [52]. An attractive simplified model for such tissue-specific binding is that nucleosomes by default cause DNA to be inaccessible and that TF binding is too weak to access individual TF binding sites. Only in areas where a cluster with many binding sites for precisely that subset of TFs that is highly expressed in the condition will these TFs jointly outcompete the nucleosomes and create a region of DNA accessibility and TF binding, i.e. similar to the qualitative model presented in [53]. We believe that the statistical mechanics model that we have used here, might also be useful to quantitatively investigate such models of enhancer function.

## Materials and Methods

### A statistical mechanical model of competitive binding of proteins to the DNA

Based on a combination of ChIP-chip data, *in vitro* binding data, and computational analysis [12], [54], [55], we previously curated [32] a collection of 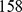 position specific weight matrices (WMs) representing the sequence-specificities of 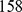 *S. cerevisiae* TFs. We let 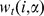 denote the WM probability that position 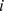 in a binding site for TF 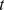 contains nucleotide 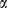. Consequently, the probability that a binding site for TF 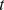 has sequence 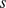 is given by
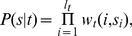
(2)where 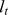 is the length of the WM for TF 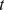 and 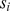 is the nucleotide at position 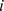 in sequence segment 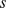. For our statistical mechanical model we wish to determine energies 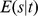 for the binding of sequence segment 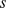 to TF 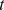. We make the standard assumption that the binding energy is a sum of individual contributions from different nucleotides in the site, i.e.
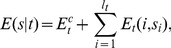
(3)where 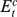 is a sequence-independent contribution to the binding energy. Under this assumption, the sequence-specific energy components 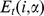 can be shown [27], [31] to be related to the WM components through
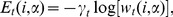
(4)where 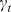 is a scale parameter, and the binding energy is expressed in units of 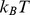.

There has been a significant amount of effort into modeling the sequence specificity of nucleosomes using data from both *in vivo* and *in vitro* experiments, e.g. [1], [15], [18], [24]. As shown in Figure 1A, different models of nucleosome sequence-specificity give predicted occupancies that are very highly correlated, and the model of [18] exhibits the most robustly high performance. We thus took the model of [18] as the basis for calculating binding energies 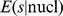 of the nucleosome to each possible 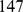 bp stretch 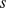. Specifically, the raw probability 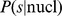 of a 147 bp long sequence segment 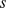 under Kaplan et al's model can be obtained using the “nucleosome_prediction.pl” script, that is provided by the authors on their website, with default parameters and using the option “raw_binding”. Using this we define a binding energy under the Kaplan model as
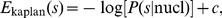
(5)In order to allow us to tune the sequence specificity of the nucleosomes, we introduce a similar scale parameter 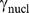 to obtain
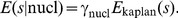
(6)Note that, at 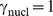, the sequence-specificity of this model will be equal to that of Kaplan et al's model, whereas at 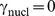 nucleosomes will have no sequence preferences whatsoever. For notational simplicity, in the following we will consider the nucleosome as just another member of the set 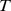 of all DNA binding factors 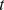.

Let 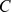 denote a (non-overlapping) configuration of TFs and nucleosomes bound to the genome and let 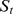 denote all segments in the genome where a binding site for factor 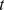 occurs. Using the standard Gibbs-Boltzmann approach, the probability of finding the cell in configuration 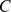 is given by
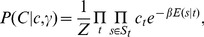
(7)where 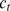 is the concentration of TF 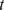, 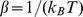 is the inverse temperature, and 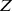 is the partition function
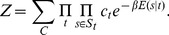
(8)Note that the probability depends on the scale factors 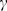 through the dependence of the binding energies 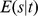 on the scale factors.

Note that, since we will be fitting the scale factors 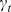, we can define
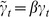
(9)and fit the 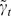. For notational simplicity, we will drop the tilde and refer to these rescaled gammas as simply 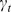. Note that this is equivalent to measuring the energy in units of 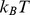.

Using only information about known binding sites, i.e. the WM entries 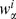, we cannot determine the sequence-independent contribution 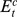 for each TF, which essentially controls how generally ‘sticky’ the TF is to DNA. To allow the comparison of binding energies of different TFs on a common scale we set 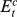 such that, in the limit of low TF concentrations, each TF has equal binding to the yeast genome. Specifically, we set 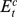 such that the average 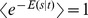, when averaging over all sequence segments 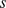 in the genome.

**Figure 8:**
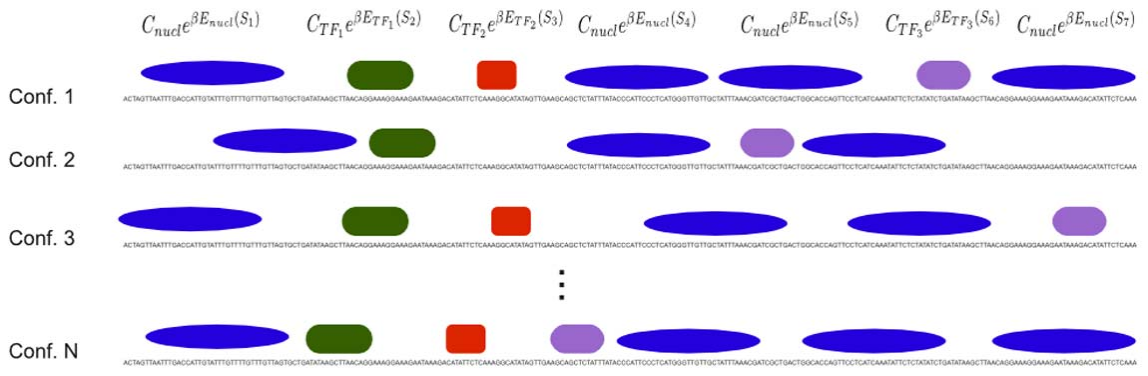
Illustration of example configurations of proteins bound to DNA. The top line indicates contributions from the individual binding sites to the overall probability of the configuration. Note that for illustration purposes, the sizes of TFs and nucleosomes are not shown to scale, e.g. the sizes of nucleosome footprints are much larger in reality.

Using this reparametrization the probability of a configuration becomes simply
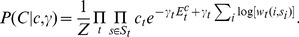
(10)
Figure 8 shows a cartoon illustrating various configurations 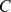 and the factors contributing to their probabilities.

The partition function can be calculated efficiently using recursion relations variously known as transfer matrices or dynamic programming, and this has been routinely used in the field to sum over non-overlapping configurations of hypothesized binding sites, e.g. [25]–[27], [29], [30]. Let 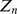 denote the partition sum for all configurations up to position 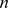 in a given chromosome. We then have
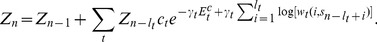
(11)Similarly, we can calculate the ‘backward’ partition sums 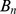 from position 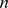 to the end of the chromosome. Finally, the probability that a binding site for factor 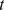 covers positions 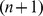 through 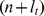 is given by
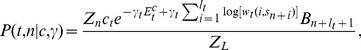
(12)where 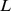 is the chromosome length. The occupancy of factor 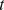 to position 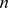 is then given by 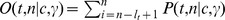. Thus, given a set of scale factors 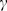 and concentrations 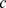, we can efficiently calculate the occupancies of all 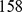 TFs and the nucleosomes across the entire yeast genome.

### Experimentally determined positions of nucleosomes and linkers

To compare the ‘raw’ occupancies as predicted by various models of nucleosome specificity and measured across several *in vivo* and *in vitro* experiments, we first downloaded the per base occupancy predictions provided by [18] and [24] and used these predicted occupancies directly. We also obtained raw data from the experiments [1], [3], [18], [38], [56]. To obtain per-base nucleosome occupancies we calculated, for the ChIP-seq data, the number of reads overlapping each position and log-transformed these read counts. For the ChIP-chip data we log-transformed the chip signal. We observed that there is a very small number of positions for which sometimes aberrantly high or low signals are reported. To avoid having these outliers skew the observed correlations we removed the 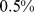 of genomic positions with highest signal and 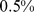 with lowest signal. We then directly calculated Pearson correlation coefficients between all data-sets and all predictions.

For the *in vivo* data, we make use of the reference map of nucleosomes and linkers for *S. cerevisiae* growing in YPD that was constructed by combining 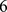 different experimental data-sets in [41]. We only retained nucleosomes that were observed in all 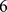 datasets and have occupancy bigger then 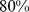 (according to the authors' annotation). This set contained 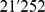 nucleosomes covering 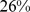 of the *S. cerevisiae* genome, and covers approximately 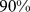 of all annotated nucleosomes in [41]. Linkers were defined as regions lying in between segments that were annotated as nucleosomes in any of the 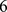 data-sets. This set contained 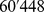 linkers covering 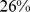 of the *S. cerevisiae* genome. As observed in [41] the distribution of linker lengths is bimodal and we separately considered ‘short linkers’ (less than 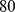 bps long) and ‘nucleosome free regions’ (longer than 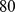 bps) in our analysis. There were 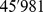 short linkers and 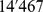 nucleosome free regions, covering 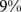 and 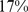 of the genome, respectively. We also separately considered the quality of the predicted nucleosome positions in promoter regions, defined as running from 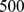 bps upstream to 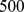 bps downstream of the TSS for each gene. The TSS definitions, as well as the definitions of the 3′ ends of genes, were taken from [57].

To assess the reproducibility of annotated nucleosome positions across the 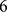 experimental data-sets we calculated, for every nucleosome in the reference annotation, the standard-deviation in the positions of the associated annotated nucleosomes in each of the 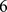 data-sets. To compare the reproducibility of the annotated nucleosomes with what may be expected by chance, given the annotation procedure, we created randomized data-sets in which each sequencing read is mapped to a randomly chosen location in the genome. We then applied the same annotation procedure to this randomized data and calculated standard-deviations of the positions of annotated nucleosomes in the same way.

We constructed a reference map of *in vitro* nucleosome positioning using 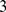 independent data-sets from [18], [19], [58] using a procedure analogous to the one used in [41]. To annotate nucleosomes for every data-set we first run the GeneTrack software [59] using parameters 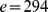 (width of the exclusion zone corresponding to configurations with non-overlapping nucleosomes), 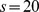 (width of the smoothing gaussian kernel), 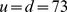 (half-width of the peak) and 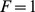 (cut-off for peak height). The values of parameters 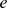 and 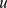 and 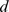 are dictated by the 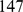 bp width of the nucleosome footprint. Since the width 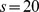 of the smoothing kernel is much smaller than the nucleosome width, the final nucleosome annotation is insensitive to the precise width of this kernel. Similarly, raising the cut-off 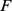 by 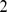-fold or 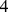-fold would only slightly reduce the number of called nucleosomes (i.e. 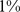 and 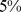 respectively) and not substantially affect the results presented in the paper. We use the annotated nucleosomes as input to GeneTrack (with the same settings), i.e. as if each annotated nucleosome were a read, to produce annotated reference nucleosomes. We retained the roughly 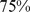 of annotated reference nucleosomes that occur in all 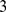 data-sets, leaving 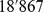 reference nucleosome genome-wide. Reference linkers were defined as regions not covered by nucleosomes in any of the annotations. There were 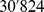 such linkers genome-wide.

### Assessing the match between predicted nucleosome coverage and experimental nucleosome positioning

To compare the experimentally annotated linker and nucleosome regions with the predicted nucleosome coverage we proceeded as follows. For a given set of parameters, i.e. concentrations 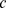 and scale parameters 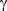, we first calculate the median of the predicted nucleosome occupancy across each annotated linker and nucleosome region. Given a critical median occupancy level 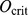, we then classified each region as either ‘nucleosome’ 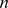 when its median occupancy was larger than 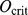 and ‘linker’ 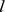 when its median occupancy was less than or equal to 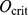. We then determined the fraction of regions both predicted and annotated as nucleosome 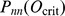, the fraction of regions predicted as nucleosome and annotated as linker 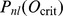, the fraction of regions predicted as linker and annotated as nucleosome 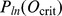, and the fraction both predicted and annotated as linkers 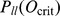. Using these we determined the *mutual information* between the predictions and the annotations based on the experimental data:
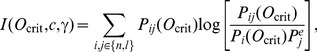
(13)where 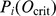 is the fraction of all regions predicted as 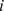, 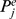 is the fraction of regions annotated as 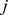, and we have explicitly indicated that this mutual information depends on the concentrations 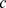 and scale factors 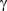 used in the predictions. We then define the mutual information 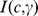 as the maximal mutual information that can be obtained varying the critical occupancy 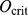, i.e.
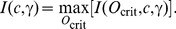
(14)Finally, to normalize the mutual information on a more intuitive scale, we divide by the maximal possible mutual information, i.e. the entropy of the experimentally observed distribution:
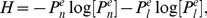
(15)to obtain
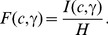
(16)Thus, 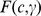 is the fraction of the information regarding nucleosome and linker positioning that is captured by the predictions, which we refer to as the *quality score*. We calculate the mutual informations 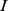 and quality score 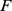 in an entirely analogous manner when considering a particular subset of experimentally annotated nucleosomes and linkers, i.e. excluding short linkers and/or focusing only on promoter regions.

To obtain predicted nucleosome coverage distributions we simply calculate the predicted occupancy at each position in the genome as described above. To obtain nucleosome coverage distributions from different experimental data-sets we proceeded as follows. As has been observed previously [20], especially for ChIP-seq data-sets, the variance in read coverage along the genome is too large to be consistent with the known overall nucleosome coverage of roughly 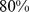. Consequently, a naive normalization in which one assumes read-coverage to be directly proportional to nucleosome occupancy would lead to unrealistically low overall nucleosome coverage. To address this, we normalize the data by rescaling log read-coverage, similar to the normalization procedure we developed previously for next-generation sequencing data [60].

Specifically, for ChIP-chip data (from a tiling array with 4 bp resolution) we obtain a signal 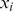 corresponding to the log-ratio of signal from the nucleosome and background sample for each probe 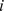 along the genome. Similarly, for ChIP-seq data we extend each read to length 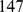 bp and defined the ‘signal’ 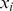 at each genomic position 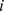 as the logarithm of the number of reads overlapping position 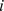. We assume that the signal 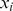 is *proportional* to the logarithm of the probability 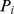 that a nucleosome is bound to the corresponding segment in the genome, i.e
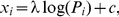
(17)where 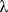 and 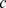 are unknown constants. We determine 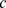 and 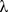 by demanding that the *average* coverage probability matches the experimentally observed average nucleosome coverage of 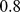, and that all coverage probabilities 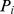 must lie in the interval 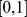. Finally, there is a small number of probes (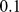 percent of all probes) with an abnormally high signal 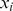 and we removed these outliers before fitting 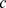 and 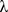. As shown in Figure S1 in Text S1, this procedure leads to highly similar coverage distributions for different data-sets.

Predicted average nucleosome coverage profiles around transcription starts and ends were obtained by simply averaging the predicted nucleosome coverage at different positions relative to TSS and transcription end over all genes. We similarly averaged the experimental coverage profiles relative to transcription starts and ends.

### Model fitting

To optimize the concentration and specificity scaling parameters 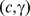 we used the Melder-Mead algorithm in combination with a simulated annealing algorithm that is implemented in the GNU Scientific Library (GSL). To avoid over-fitting when fitting different models with varying numbers of parameters we used a 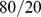 cross-validation scheme for each model and data-set. That is, for each data-set and model, we randomly divide the data-set of annotated nucleosomes and linkers into 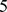 equally sized sub-sets. We then perform the parameter fitting 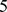 independent times, each time optimizing the parameters on 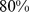 of the data and then evaluating the final quality score of the model on the ‘test-set’ containing the remaining 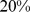 of the data. Whereever quality scores are shown we show the average quality score and its standard-error across the 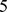 test-sets.

For the *in vivo* reference set of nucleosomes and linkers, we first performed optimizations of the nucleosome-only model with different (fixed) values of the specificity scaling parameter 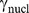, i.e. optimizing only the concentration 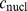. For both the *in vivo* and *in vitro* reference sets we optimized the two-parameter nucleosome-only model (obtaining an optimal 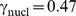 for the *in vivo* data, and 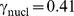 for the *in vitro* data). After this we fixed the nucleosome specificity and concentration to their optimal values and, for the *in vivo* data, fitted the model with all TFs, fitting the concentrations and scale parameters for all TFs.

For the biophysical characterization of the fitted model, we first averaged the fitted concentrations 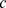 and scale parameters 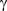 over the 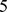 training sets. We then calculated the predicted posterior binding probabilities 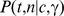 for every factor 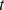 (i.e. the nucleosomes and all TFs) at every position 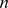 in the yeast genome. For each factor 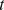, we then calculated the fraction of the genome 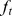 covered by this protein: 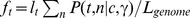, where 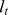 is the length of the footprint of protein 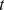 and 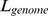 is the length of the yeast genome. We also calculated the average binding energy 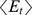 of the binding sites of each protein 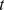, i.e. 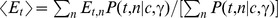, and its standard deviation 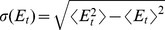. Here 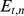 is the binding energy of protein 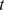 at position 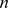, measured in units 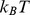. Finally, we calculated the average entropy 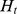 per binding site:
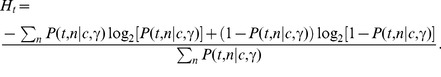
(18)


To calculate the information content for a TF 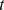, as shown in Figure S11 of Text S1, we used the standard formula
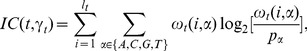
(19)where the 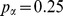 are background probabilities (which we chose uniform) and the 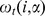 are the weight matrix entries. Note that, to incorporate the scaling parameter 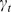, the weight matrix entries are rescaled according to:
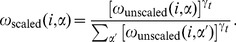
(20)


To assess the contribution of different TFs we fitted, for each TF, the model with nucleosomes and this single TF. For each TF we calculated, on each of the 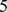 test-sets, the difference 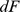 between the quality score using only the nucleosome, and the quality score with the TF added, and determined the mean 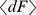 and standard error 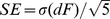 over the 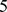 test-sets. We then ranked the TFs by the 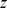-statistic 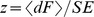. These fits and statistics were obtained separately for both the *in vivo* and the *in vitro* data. Finally, we also created a set of 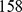 randomized WMs by, for each WM, randomly shuffling the columns of the WM. Note that this randomization conserves both the sequence composition and the information scores of the WMs. We then performed the fitting with these 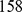 randomized WMs and obtained 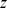-statistics in the precise same way.

For the *in vivo* data we then also fitted models including the top 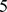, 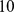, 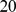, and 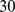 TFs from the list ranked by their 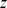-statistic, re-optimizing all parameters. Finally, to assess the contribution of the nucleosome specificity when TFs are added for the *in vivo* data, we fitted the model including all TFs, but without nucleosome sequence specificity, i.e. setting 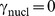.

### Annotating chromatin related TFs

To annotate TFs with known roles in chromatin dynamics we used the Gene Ontology (GO) annotations available from the Saccharomyces cerevisiae genome database. We considered a TF ‘chromatin related’ when its GO annotation included any of the following categories:

- GO:0016568 chromatin modification.
- GO:0006338 chromatin remodeling.
- GO:0008301 DNA bending activity.
- GO:0031491 nucleosome binding.
- GO:0003682 chromatin binding.
- GO:0033698 Rpd3C(L) A histone deacetylase complex which deacetylates histones across gene coding regions.

Finally, we also added the TFs identified in [12] to this list. To calculate the over-representation of ‘chromatin related’ TFs among the top 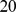 TFs effecting nucleosome positioning, we performed a simple hypergeometric test.

### Protein-protein interactions between TFs, histones, and chromatin remodelers

We first annotated yeast proteins that are either (1) part of chromatin remodeling complexes, (2) histone modification enzymes, or (3) histones themselves. Subunits of chromatin remodeler complexes were taken from [61], [62]. As subunits of histone modification enzymes we took genes that have GO annotation “covalent chromatin modification” and all children GO categories, i.e. histone methylation, acetylation etcera (108 genes in total). Information about protein-protein interactions were downloaded from the STRING database (http://www.string-db.org, file ‘protein.links.detailed.v9.0.txt.gz’), using only experimental evidence with a cutoff of 400. After determining all known protein-protein interactions between the 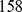 TFs and the three classes of proteins (histones, histone modification enzymes, and subunits of chromatin remodeling complexes) we calculated enrichment of interactions between each class and the top 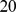 TFs that significantly explain nucleosome positioning. To assess the significance of the enrichment we used a simple hypergeometric test. The results are listed in Table 1.

## Supporting Information

Table S1 Information for every transcription factor about Z-scores, fitted parameters and protein-protein interactions with chromatin remodeling complexes, histone modification enzymes and histones.
(XLS)

Text S1 Supplementary file. This pdf file contains the supplementary Figures S1, S2, S3, S4, S5, S6, S7, S8, S9, S10, S11. The supplementary Table S1 is provided in separate excel file.
(PDF)
