# Supplementary material for: Nucleosome Free Regions in Yeast Promoters Result from Competitive Binding of Transcription Factors That Interact with Chromatin Modifiers
Source: PLoS Comput Biol. 2013 Aug 22;9(8):e1003181. doi: 10.1371/journal.pcbi.1003181 (PMC3749953; doi:10.1371/journal.pcbi.1003181)
Supplement: Text S1 — Supplementary file. This pdf file contains the supplementary Figures S1, S2, S3, S4, S5, S6, S7, S8, S9, S10, S11. The supplementary Table S1 is provided in separate excel file. (PDF) [file pcbi.1003181.s002.pdf]

Nucleosome free regions in yeast promoters result from competitive binding of transcription factors that interact with chromatin modifiers

Evgeniy A. Ozonov[1] and Erik van Nimwegen[1,*]
1 Biozentrum, University of Basel, and Swiss Institute of Bioinformatics, Basel, Switzerland.
∗ Corresponding Author: Erik van Nimwegen, erik.vannimwegen@unibas.ch.

Supplementary file

This pdf file contains the supplementary Figures S1-S11.
The supplementary Table S1 is provided in separate excel file.

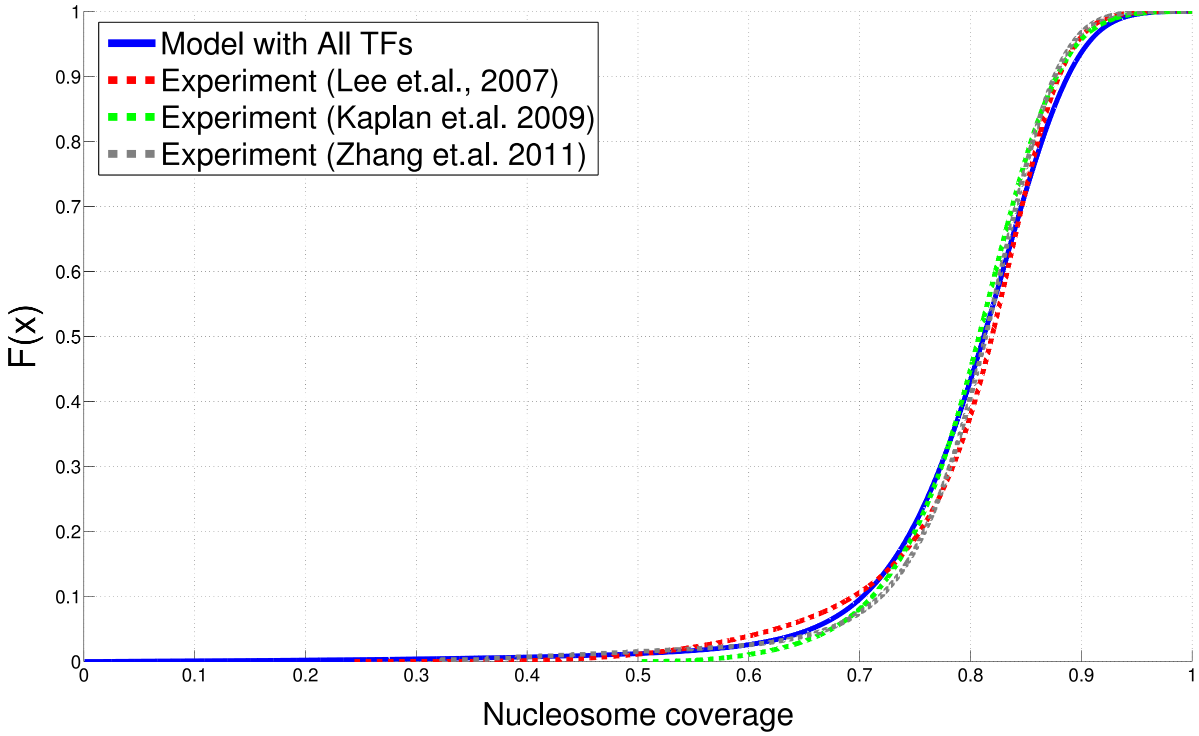

Figure S1. Comparison of nucleosome occupancy distributions across three experimental data-sets and the model including all TFs. Cumulative distributions of nucleosome occupancies as measured in [1] (red dotted curve), as measured in [2] (green dotted curve), as measured in [3] (grey dotted curve), and as predicted by the model including all TFs (blue curve).

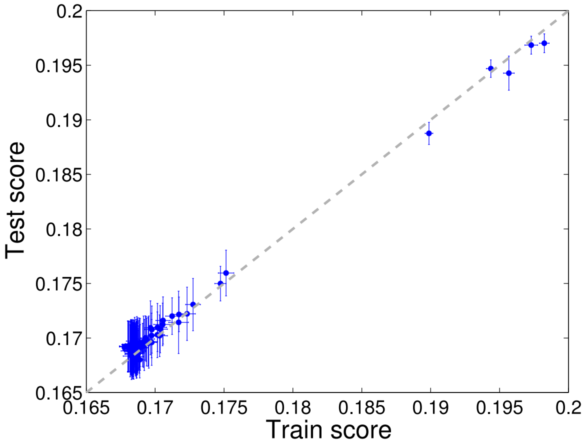

Figure S2. Comparison of the model's training and test scores shows there is no over-fitting. For each of the 158 yeast TFs, we fitted a model containing a single TF plus nucleosomes to the *in vivo* reference map of nucleosomes and linkers genome-wide, optimizing the quality score F. We ranked all TFs by the z-statistic they attain and similarly fitted models that contain the nucleome plus the top 5, top 10, top 20, top 30, and all TFs. For each model we used 80/20 cross-validation, i.e. we fitted the model on 80% of the data and than evaluated it on the test-set of the remaining 20%. We performed this fitting 5 times and then calculated both the mean and standard-deviation of the quality score F obtained on both the training and test-sets. The figures shows a scatter of the quality scores on the training and test-sets for each of the models fitted. The error-bars denote the standard-error across 5 repeats. The figure shows that, although the test-set scores tend to vary more than the training set scores, the test scores are not consistently lower than the training scores, i.e. the model does not show any over-fitting.

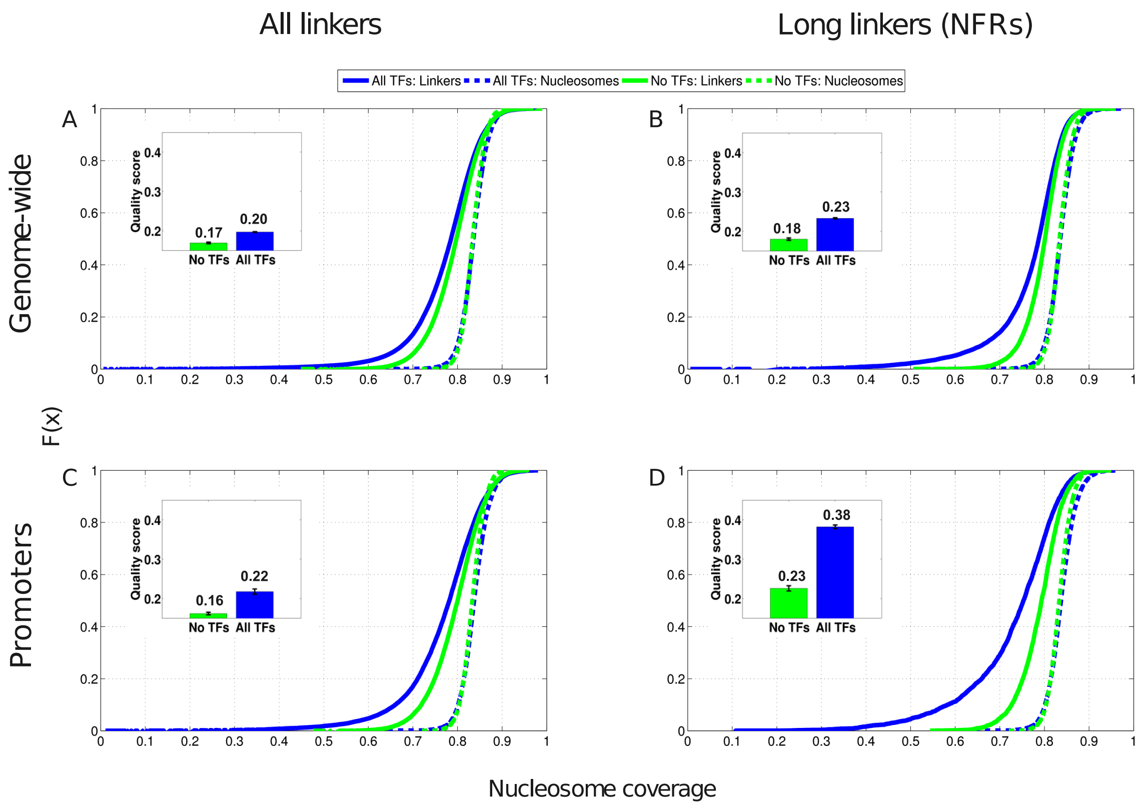

Figure S3. Quality of the predicted nucleosome positioning profiles when including competition with TFs. The insets in each panel show the quality scores F of the model both including TFs (blue bar) and without TFs (green bar) in predicting annotated nucleosome and linker positions. The error-bars indicate the standard-error across 5 test sets. The curves in each panel show the cumulative distributions of predicted nucleosome coverage in annotated nucleosomes (dotted lines) and annotated linkers (solid lines) for the model using only nucleosomes (green) and the model including TFs (blue). **A**: Predicting all annotated linkers and nucleosome genome-wide. **B**: Predicting annotated nucleosomes and nucleosome free regions (long linkers) genome-wide. **C**: Predicting annotated nucleosomes and linkers in promoter regions. **D**: Predicting annotated nucleosomes and nucleosome free regions (long linkers) in promoter regions. Note that, for all 4 data-sets, inclusion of the TFs has very little effect on the coverage distribution observed at nucleosomes (i.e. annotated nucleosomes are generally predicted to be highly occupied) but that the TFs significantly lower the predicted coverage at annotated linkers, especially the long linkers in promoters.

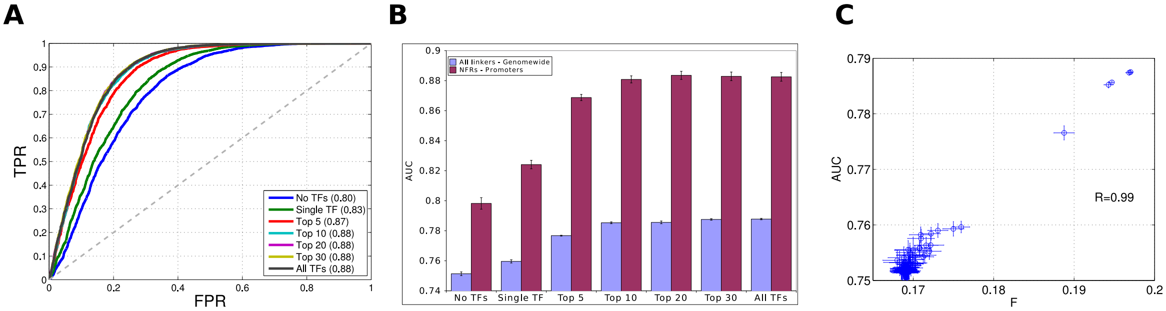

Figure S4. Comparison of quality scores F with model assessment based on ROC curve analysis. **A:** For the models with no TFs, the top 1, 5, 10, 20, 30, and all TFs, we obtained a ROC curve for the classification accuracy of the model, i.e. by varying a cut-off in the predicted nucleosome coverage we calculated the rate of true-positive and true-negative prediction of nucleosomes/linkers. Similarly to the results obtained with the F quality score, the area under the curve (AUC) increases rapidly when the first few TFs are added and the performance saturates after $10 - 20$ TFs are added. **B:** Performance as measured by AUC for the models with increasing numbers of TFs, both for all linkers genome-wide (blue bars), as well as long linkers (NFRs) at promoters (red bars). Apart from a change in scale, the results look virtually identical to those obtained with the quality score F. **C:** A scatter of the quality score F against the AUC for all fitted models shows that the two measures of performance are very highly correlated.

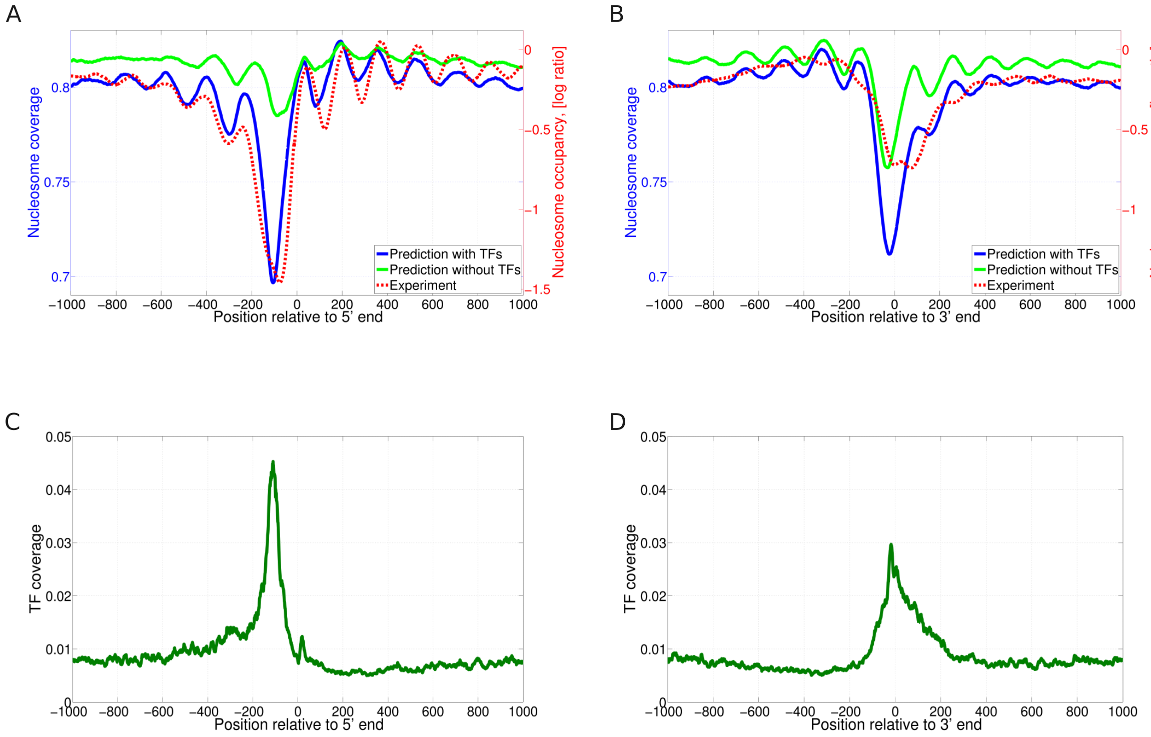

Figure S5. Nucleosome and TF coverage profiles around starts and ends of genes. **A:** Averaged nucleosome coverage near the transcription starts. **B:** Average nucleosome coverage near the ends of genes. Each curve shows the average nucleosome coverage at different positions relative to transcription start or end averaged over all genes. Red dashed lines correspond to experimentally measured nucleosome coverage (data from [1], right vertical axis). The solid lines correspond to the predicted nucleosome coverage by the model including only nucleosomes (light green) and the model including all TFs (blue), left vertical axis. **C:** Averaged TF coverage (summed over all 158 TFs) relative to transcription start sites. **D:** Average TF coverage near transcription ends.

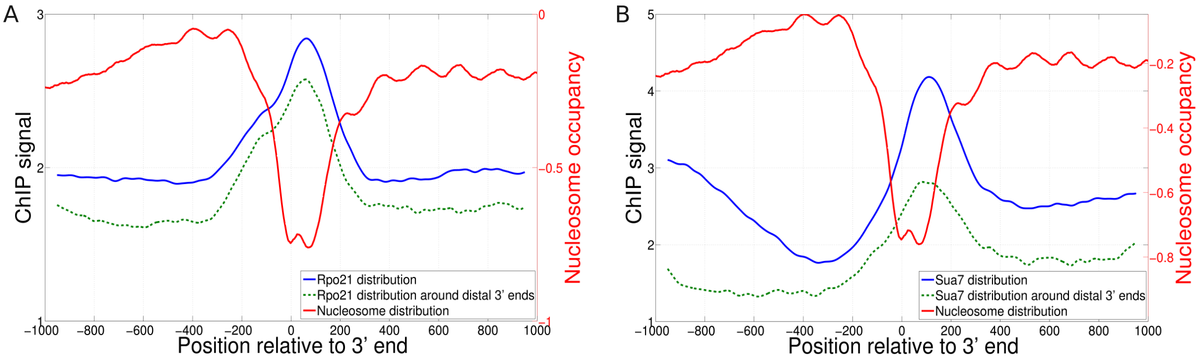

Figure S6. Comparison of nucleosome coverage around ends of genes with binding profiles of RNA polymerase subunits. **A:** Average binding profile of the RNA polymerase II sub-unit Rpo21 around 3' ends of genes. **B:** Average binding profile of the general transcription factor Sua7 around 3' ends of genes. The blue curve corresponds to the average ChIP signal (log-ratio, left vertical axis) at each position from 1000 bps upstream to 1000 bps downstream of transcription end. The green dashed line shows the average ChIP signal when only genes whose ends are distal to the next transcription start are included. For reference, the red curves show the experimentally observed nucleosome coverage profiles (data from [1], right vertical axis). The results indicate that Rpo21 and Sua7 are observed to bind precisely in the region corresponding to the 3' nucleosome depleted region. The fact that the binding profiles look similar for 3' ends of genes that do not have a neighboring transcription start site nearby shows that the Rpo21 and Sua7 binding is not associated with a nearby promoter.

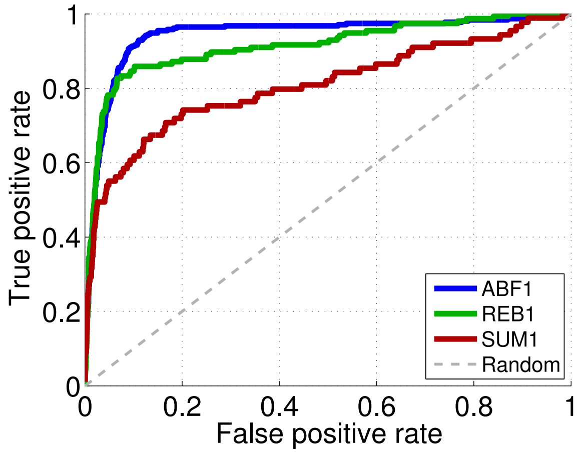

Figure S7. Performance of the model with the nucleosome and all TFs in predicting the observed target promoters of the TFs Abf1, Reb1, and Sum1. The ChIP-chip binding data of [4] reports, for each TF, which promoter regions are bound by the factor and we used these as a reference set to compare with our predictions. For each promoter and each TF, we calculated a total 'target score' for the model by summing the predicted posterior probabilities of binding across all positions in the promoter. We then obtained ROC curves by varying a cut-off on this 'target score'. The figure shows the ROC curves of True positive and False positive rates obtained. Although our model was only optimized to fit observed nucleosome positioning, we see that it also accurately predicts the target promoters of these three TFs.

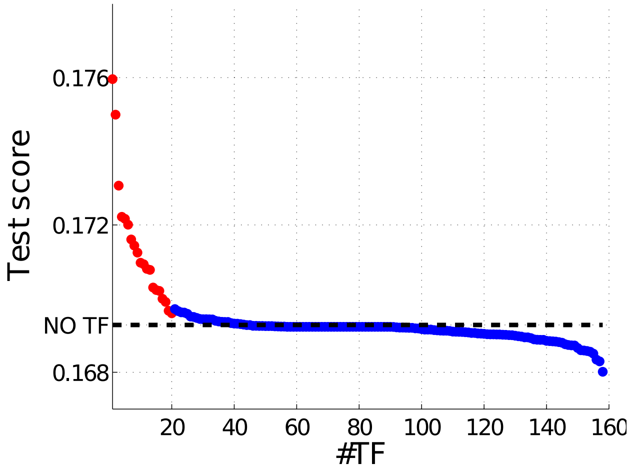

Figure S8. Distribution of test scores for the models with nucleosomes and a single TF. For each TF we fitted the model including nucleosome specificity and the single TF on the training set and then determined the quality score (fraction of explained information) on the test set of annotated nucleosomes and linkers. We sorted TFs by their quality score and the figure shows the quality score as a function of TF number in this sorted list. For reference, the quality score obtained with the model without any TFs, i.e. nucleosome specificity only, is shown as a black dashed line. Note that the majority of TFs do not improve the quality score over the nucleosome-only model. The quality scores of the top 20 TFs are indicated in red.

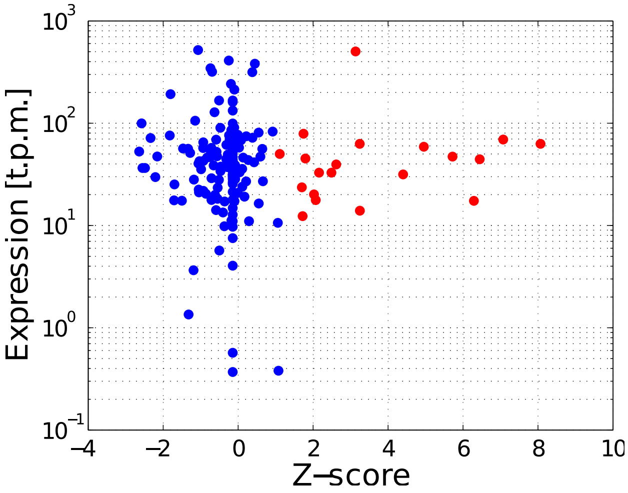

Figure S9. Relation between TF significance for explaining nucleosome positioning and mRNA expression levels in YPD. For each TF a z-statistic was calculated (see Materials and Methods) that quantifies the extent to which the TF contributes to explaining nucleosome positioning genome wide. For each TF the z-statistic is shown on the horizontal axis against the TF's mRNA expression level in YPD expressed in tags per million (vertical axis, data from [5], note that the method smsDGE described in [5] does not require normalization by transcript length). Red dots correspond to the 20 TFs that most significantly contribute to nucleosome positioning. The figure shows that there is little correlation between expression level and the z-statistic.

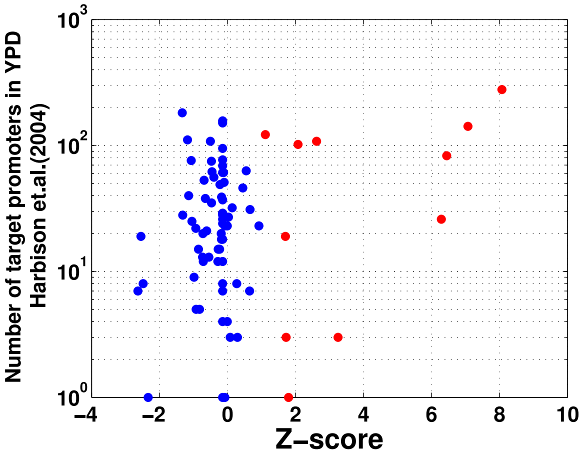

Figure S10. Relation between the total number of promoters with binding in YPD and the significance in explaining nucleosome positioning of TFs. For each TF a z-statistic was calculated (see Materials and Methods) that quantifies the extent to which the TF contributes to explaining nucleosome positioning genome wide. For each TF the z-statistic is shown on the horizontal axis against the total number of promoters that have binding of the TF in YPD (p-value < 0.05) as measured by [4]. The top 20 TFs are indicated in red. There is no clear correlation between the total number of target promoters in YPD and the z-statistic.

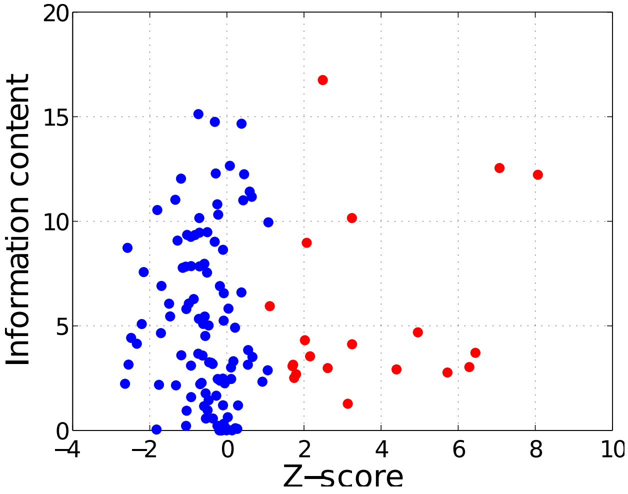

Figure S11. Relation between the information content of each TF's binding motif and its significance in explaining nucleosome positioning. For each TF a z-statistic was calculated (see Materials and Methods) that quantifies the extent to which the TF contributes to explaining nucleosome positioning genome wide. For each TF the z-statistic is shown on the horizontal axis against the information content (in bits, vertical axis) of its binding motif (i.e. a position specific weight matrix). Note that the information content calculation takes into account the binding specificity factor γ_t that is fitted for each TF t. The top 20 most significant TFs are indicated in red. Note that there is no correlation between information content and z-statistic.

References

1. Lee W, Tillo D, Bray N, Morse RH, Davis RW, et al. (2007) A high-resolution atlas of nucleosome occupancy in yeast. Nat Genet 39: 1235–1244.

2. Kaplan N, Moore IK, Fondufe-Mittendorf Y, Gossett AJ, Tillo D, et al. (2009) The DNA-encoded nucleosome organization of a eukaryotic genome. Nature 458: 362–366.

3. Zhang Z, Wippo CJ, Wal M, Ward E, Korber P, et al. (2011) A packing mechanism for nucleosome organization reconstituted across a eukaryotic genome. Science 332: 977–980.

4. Harbison CT, Gordon DB, Lee TI, Rinaldi NJ, Macisaac KD, et al. (2004) Transcriptional regulatory code of a eukaryotic genome. Nature 431: 99-104.

5. Lipson D, Raz T, Kieu A, Jones DR, Giladi E, et al. (2009) Quantification of the yeast transcriptome by single-molecule sequencing. Nat Biotechnol 27: 652–658.
